# 眼部慢性移植物抗宿主病的治疗

**DOI:** 10.3760/cma.j.issn.0253-2727.2022.10.015

**Published:** 2022-10

**Authors:** 先静 程, 容娣 袁, 曦 张

**Affiliations:** 1 陆军军医大学新桥医院血液病医学中心，创伤、烧伤与复合伤国家重点实验室，全军血液病中心，重庆市医学重点学科，重庆 400037 Medical Center of Hematology, Xinqiao Hospital of Army Medical University, State Key Laboratory of Trauma, Burns and Combined Injury, PLA Blood Disease Center, Chongqing Key Discipline of Medicine, Chongqing 400037, China; 2 重庆大学医学院，重庆 400030 School of Medicine Chongqing University, Chongqing 400030, China; 3 陆军军医大学新桥医院眼科，重庆 400037 Ophthalmology Department, Xinqiao Hospital of Army Medical University, Chongqing 400037, China

慢性移植物抗宿主病（cGVHD）是异基因造血干细胞移植（allo-HSCT）后的常见并发症，发生率为30％～70％[1]。cGVHD类似于自身免疫性疾病，涉及包括皮肤、肺、肝、胃肠道、口腔、生殖器和眼睛等多个部位。眼部GVHD（oGVHD）的发生率为40％～60％，临床表现轻、重程度不一，但如果不及时治疗和控制，高达60％～90％的患者出现视力下降，严重影响患者日常生活与工作[2]–[3]。oGVHD的发病机制十分复杂，尚未完全明晰。目前主要认为与T细胞浸润引发的多种免疫反应相关，包括免疫细胞募集、产生炎性细胞因子和趋化因子、诱导细胞凋亡等[4]。迄今，oGVHD尚无统一的诊断标准和治疗指南，但是又非常影响移植患者的生存质量，常常成为患者就医的主诉甚至引起心理障碍。

一、临床表现

oGVHD的临床表现比较复杂，可累及整个眼表系统的所有结构，其中主要影响患者眼前节的结构（泪腺、睑板腺、角膜和结膜）。根据美国国家卫生院（NIH）发布的cGVHD诊断标准，oGVHD的临床特征包括眼新发干燥、“沙砾感”、疼痛感、瘢痕性结膜炎、干燥性角结膜炎（KCS）和点状角膜病，其他特征还包括畏光、眶周色素沉着和眼睑炎[1]。严重的oGVHD还可能出现角膜溃疡、角膜穿孔，角膜新生血管形成等，严重影响患者视力。

二、诊断

oGVHD的诊断尚缺乏统一标准，现在主要根据NIH诊断和分期工作组颁布的诊断标准，即非其他原因引起的Schirmer试验平均值≤5 mm/5 min或Schirmer试验平均值≤6～10 mm/5 min且通过裂隙灯检查新发KCS和至少有一个其他器官有额外的独特表现[1]。另一国际共识小组（ICCGVHD）则建议oGVHD的诊断需综合眼表疾病指数（OSDI）、Schirmer Ⅰ试验、角膜荧光素染色（CFS）和结膜充血四个指标进行分析[5]。然而，上述症状和体征缺乏特异性，无法与其他原因引起的干眼相鉴别。目前，oGVHD的诊断主要根据相关眼部临床表现及眼科检查来确定，但其临床表现常出现在疾病发展中后期，而侵入性眼科检查会破坏患者眼表结构，不利于长期随访。因此，临床上亟需早期诊断和非侵入性的客观特异诊断方式。

三、治疗

oGVHD的治疗目标主要是缓解症状、眼部润滑、控制泪液蒸发和引流、减少眼表炎症，严重、顽固或复杂的病例可能需要手术治疗。

（一）全身治疗

由于oGVHD是cGVHD全身病变的一部分，因此oGVHD的治疗需要考虑是否采用全身免疫抑制疗法，但并非所有的病例均需进行全身治疗，如对于只累及眼部的轻度cGVHD可以只开展局部治疗而无需全身治疗；且对于需要全身治疗的患者，眼部症状的缓解程度也不一定和全身治疗反应一致。目前，cGVHD全身治疗药物首选糖皮质激素联合或不联合钙调磷酸抑制剂[6]。对于糖皮质激素难治性GVHD患者，可以采用二线治疗方法[7]–[8]。GVHD患者眼部症状对于体外光化学疗法、利妥昔单抗、西罗莫司、霉酚酸酯的反应率分别为43％、31％、60％、33％[9]。此外，细胞治疗cGVHD也有广泛应用前景[10]。继发于cGVHD的难治性干眼症患者持续接受间充质干细胞静脉输注3个月后，全部22例患者中12例（54.55％）眼部症状减轻，OSDI评分和Schirmer试验结果均有改善[11]。

尽管全身治疗可能会整体改善oGVHD，但对于局部效果还有局限性：①无法充分润滑眼表，而oGVHD需要局部润滑剂，尤其是严重干眼症的患者；②与局部给药相比，全身治疗获得的眼表药物浓度较低。因此，在oGVHD治疗选择中局部给药途径是必要的。

（二）局部药物治疗

1. 人工泪液：人工泪液是一种已被证实的必不可少的一线治疗方法，对眼表有湿润作用。一项大型回顾性研究分析比较了1293例患者使用不同类型人工泪液产品情况，结果显示不同产品没有明确的统计学差异[12]。需要注意的是，在选择人工泪液种类时，应避免选择含有防腐剂和磷酸盐的制剂，因为当用于炎症或角膜受损的眼睛时，防腐剂对眼表上皮细胞具有细胞毒性，而含磷酸盐的滴剂可能会导致钙化，在角膜表面形成不溶性结晶沉积物。

2. 血清类眼用制剂：血清类眼用制剂包括自体血清、脐带血清等。较人工泪液而言，血清类制剂富含各种生长因子和细胞因子，可以刺激细胞增殖和迁移，在眼表伤口愈合中起关键作用。

（1）自体血清：与人工泪液相比，自体血清的成分更似于天然人泪液，且其维生素A、转化生长因子β1（TGF-β1）、神经生长因子（NGF）、纤维连接蛋白和溶菌酶等浓度更高，能更好地改善眼表疾病的症状和体征[13]。Tahmaz等[14]曾用100％自体血清滴眼液治疗严重oGVHD患者：治疗6个月后，患者角膜染色（右眼：3±1.03降至2±1.43，*P*＝0.004；左眼：4±1.0降至2±1.09，*P*＝0.001）和眼部症状（OSDI：88±20.59降至63±22.77，*P*＝0.02）改善，视力也有所提高，且无不良反应。然而，如果oGVHD患者有全身感染性疾病或其他不适宜制备自体血清的全身疾病时，脐带血清、同种异体血清或其它眼用生物制剂可能是更好的治疗选择。刘靖等[15]曾报道需长期使用促上皮修复治疗的oGVHD患者，可考虑使用小牛血去蛋白提取物眼用凝胶替代自体血清。

（2）脐带血清：与自体血清相比，脐带血清中TGF-β、NGF和P物质等浓度更高[13]。Yoon等[16]曾比较20％自体血清和脐带血清滴眼液治疗oGVHD严重干眼症的疗效：接受脐带血清治疗1个月和2个月后，患者的眼部症状和角膜上皮病变评分更低。从临床角度来看，脐带血清疗法也比自体血清疗法更具有优点：一次分娩可获得大量脐带血清，无需反复抽取患者血液；脐带血清含有具有抑菌作用的IgG、溶菌酶和补体等成分。但自体血清与脐带血清都存在保存困难、污染风险等问题。

3. 局部免疫抑制及抗炎制剂：（1）糖皮质激素滴眼液：局部类固醇可提供较高眼表药物浓度，适用于眼部炎症明显的oGVHD患者。一项回顾性分析研究显示：7例oGVHD患者接受1％醋酸泼尼松治疗7周后均完全缓解，但3例患者双眼出现糖皮质激素诱导的高眼压[17]。而另一研究则显示oGVHD患者对低剂量局部糖皮质激素反应较差：接受0.5％氯替泼诺滴眼液持续治疗4周后，oGVHD患者OSDI由59.2±6.7增至61.1±7.1（*P*＝0.66），CFS由5.5±0.5减至5.3±1.1（*P*＝0.85）[18]。糖皮质激素局部长期使用可导致眼压升高和感染，可能造成严重的非GVHD眼部并发症。

（2）钙调磷酸酶抑制剂滴眼液：环孢素A是克服糖皮质激素不良反应的一种替代选择，适用于无明显炎症活动，但存在潜在炎症过程的干眼症患者。Rao等[19]曾用0.05％环孢素A滴眼液治疗8例oGVHD干眼症患者，其中7例患者症状得到改善。治疗3个月后，Schirmer试验评分（*P*＝0.003）、泪液破裂时间（*P*＝0.002）和泪液溶菌酶水平（*P*＝0.033）方面有统计学意义的改善。但笔者认为，尽管局部应用环孢素A对oGVHD的有益作用已在一些研究中得到证实，但仍需要大型随机研究数据进行验证。

他克莫司也是一种从链霉菌中提取的大环内酯类免疫抑制剂，效果是环孢素A的近100倍。Jun等[20]用0.02％他克莫司软膏持续治疗oGVHD患者≥6个月：在治疗2～8周后，患者眼表炎症评分从2.8降至0.6（*P*＝0.001）、炎症加重的频率和需要糖皮质激素治疗的次数也相应减少；唐旭园等[21]曾比较0.1％他莫克司滴眼液与玻璃酸钠滴眼液治疗oGVHD干眼症疗效：治疗2个月后，除角膜荧光素染色，患者的视力、OSDI评分、泪液分泌试验、泪河高度、首次非侵入性泪膜破裂时间、结膜充血和睑板腺萎缩程度等均较玻璃酸钠治疗组改善明显（*P*<0.05）；另一项Ⅰ/Ⅱ期前瞻性随机对照临床试验还比较了外用0.05％他克莫司与0.5％甲泼尼龙的效果差异：持续治疗10周后，他克莫司治疗组（降低55％，*P*<0.001）降低CFS评分方面比甲泼尼龙组更有效（降低23％，*P*＝0.09），但他克莫司组OSDI评分（减少27％，*P*＝0.02）降低没有甲泼尼龙组显著（减少32％，*P*＝0.06）。此外，两组综合耐受性得分差异无统计学意义（*P*＝0.06），但他克莫司组的烧灼感更明显（*P*＝0.002），甲泼尼龙组眼压升高更明显（*P*＝0.04）[22]。

（三）手术治疗

严重oGVHD患者还可能发生重度干眼症、角膜溃疡甚至穿孔等，这可能需要手术治疗。

1. 泪小点栓塞术：泪小点栓塞术是一种有效的治疗手段，通过抑制泪液引流来帮助保护泪膜，从而维持眼表。主要推荐用于重度泪腺功能异常的患者。但有研究者认为泪小点栓塞可能会导致富含炎性细胞因子的泪液滞留时间延长，有进一步加重眼表炎症的顾虑。Sabti等[23]用泪小点栓塞术治疗cGVHD所致KCS，患者的主观舒适度提高（*P*<0.001）、病理性角膜荧光素染色减少（*P*<0.001），无明显不良反应且未加重患者眼部炎症，但59％的患者出现栓子自发缺失。Singh等[24]也曾报道与非oGVHD患者相比，oGVHD患者的栓子保留率显著降低（*P*<0.001）。如果出现栓子反复丢失情况，点状灼烧是一种可考虑的有效治疗手段。

2. 睑缘缝合术：睑缘缝合术可以减少泪液蒸发，主要用于慢性重度干眼患者。病例研究显示，因免疫低下感染眼部带状疱疹、角膜缘缺血严重、常规局部药物治疗无效的oGVHD患者接受了羊膜移植术、外侧睑缘缝合术和泪小点栓塞术治疗20 d后，角膜缘缺血和结膜炎症得以改善，上皮缺损的面积减小[25]。虽然通过睑缘缝合术可减少眼表的暴露，以减少泪液的蒸发，但可能会限制眼周边的视野范围，也不利于美观。

3. 角膜移植和角膜缘干细胞移植：角膜移植和角膜缘干细胞移植是治疗眼表疾病的重要手段。穿透性角膜移植术是治疗oGVHD角膜穿孔常用的治疗方法。Heath等[26]曾用穿透性角膜移植术联合角膜缘干细胞移植治疗严重oGVHD，该患者眼部整体情况得以改善。但在严重眼表疾病和泪液不足的情况下，同种异体移植物的存活率较低。板层角膜移植术是一种可以恢复眼睛完整性更安全的方法，因为免疫排斥反应、术中和术后并发症风险较低。角膜缘干细胞移植主要适用于角膜缘干细胞缺乏症和需修复受损角膜表面患者，但也存在免疫排斥风险。Meller等[27]曾用HLA相同的同种异体相关角膜缘干细胞治疗1例双侧角膜缘干细胞缺乏的oGVHD患者，持续31个月的随访发现该患者眼表重建成功，角膜表面清晰、光滑、稳定，无角膜缘干细胞缺乏复发情况。但术后9个月出现角膜小穿孔，通过角膜移植术结合去除刺激性眼睑睫毛成功治疗。

4. 羊膜移植：羊膜移植也是恢复角膜完整性的有效方法。Peric等[28]成功用羊膜移植治疗4例严重oGVHD患者，并发现治疗后患者角膜缺损处上皮化、症状缓解，且无不良反应和感染发生。Ikarashi等[29]用羊膜移植治疗oGVHD患者，9个月后患者右眼最佳矫正视力（BCVA）从20/100提升到20/25，左眼BCVA从20/32提升到20/22，但1年后右眼出现不明原因的角膜穿孔。Lavaris等[30]采用无缝线无胶水的多层羊膜修补患者眼部受影响区域（只使用绷带镜保持羊膜附着），3个月后，患者症状改善、角膜完整、角膜厚度增加，表明无缝线无胶水的多层羊膜修补可以防止角膜病变的进一步恶化。

（四）辅助治疗

此外，睑缘清洁、热敷熏蒸、睑板腺按摩也可有效改善oGVHD患者的睑板腺表达能力。其他的治疗手段还包括佩戴绷带软镜、湿房镜、巩膜镜等。

四、结语

综上所述，oGVHD的诊断评估、治疗和支持性护理是HSCT后多慢病管理的重要组成部分。患者诊治和管理应是跨学科、多模式的，并根据患者的情况和年龄进行个体调整。目前仍缺少一种完全令人满意的治疗方案，也缺乏循证级别较高的临床研究数据，需要我们进一步研究和积累；此外，oGVHD治疗效果受限的另一原因是诊断和干预的时机较晚，有些患者已经存在永久不可逆组织损伤，再修复的希望渺茫。因此，对于oGVHD提倡预防为先、早期诊断、早期干预、规范化治疗。
